# Prevalence and Drivers of Child Food Poverty in Ethiopia: Evidence From a Nationally Representative Survey

**DOI:** 10.1111/mcn.70186

**Published:** 2026-04-13

**Authors:** Adamu Belay, Ramadhani Noor, Bedasa Tessema, Meseret woldeyohannes, Nahom Tefera, Meron Girma, Alemnesh Petros, Nardos Birru, Yetayesh Maru, Stanley Chitekwe, Hiwot Darsene, kaleab Baye, Masresha Tessema

**Affiliations:** ^1^ Nutrition, Environmental Health and Non‐Communicable Disease Research Directorate, Ethiopian Public Health Institute Addis Ababa Ethiopia; ^2^ United Nations Children's Fund (UNICEF) Addis Ababa Ethiopia; ^3^ Nutrition Coordination Lead Executive Office, Ministry of Health Addis Ababa Ethiopia; ^4^ Center for Food Science and Nutrition Addis Ababa University Addis Ababa Ethiopia

**Keywords:** child food poverty, dietary diversity, Ethiopia, food systems, nutrition security

## Abstract

Child food poverty, defined as inadequate access to and consumption of a nutritious and diverse diet in early childhood, can have lasting consequences for health, development, and economic productivity. However, nationally representative data on its magnitude and factors remain scarce in Ethiopia. This study aims to estimate the prevalence of child food poverty at national and regional levels, identify its key factors, and quantify the absolute burden among children under 5 years of age. We conducted a population‐based, cross‐sectional Food and Nutrition Strategy baseline survey. Dietary intake was assessed using a 24‐h recall for children aged 6–23 months (*n* = 2,969). Proportion of severe‐ (0–2 food groups) and moderate‐ (3–4 food groups) child food poverty was calculated. Multivariable logistic regression was used to identify predictors of severe food poverty. Extrapolations were performed to estimate the absolute number of children aged 6–59 months experiencing food poverty nationally. Overall, 92% of children aged 6–23 months lived in severe or moderate food poverty, which translates to about 12 million Ethiopian children under five. Significant predictors of severe food poverty included low maternal education (AOR 1.72, 95% CI: 1.22,2.41), no antenatal care visits (AOR 1.69, 95% CI: 1.38,2.08), and lowest household wealth quintile (AOR 2.98, 95% CI: 1.85,4.68). Child food poverty is widespread in Ethiopia, with substantial regional variation and strong links to maternal education, healthcare access, and household wealth. Urgent, multi‐sectoral nutrition interventions are needed to protect young children's, strengthen public health systems, and enhance emergency preparedness.

## Summary

- In Ethiopia, approximately 12 million children under five, equivalent to 92% of this age group, experience moderate or severe child food poverty.
- Children facing severe food poverty are 40% more likely to be stunted and 28% more likely to be wasted. Tackling child food poverty through targeted, nutrition‐sensitive interventions is essential to achieving Ethiopia's goal of zero stunting by 2030.
- Severe child food poverty affects children across both poor and non‐poor households, demonstrating that factors beyond household income contribute significantly to inadequate child diets.
- Children from the poorest households are nearly three times more likely to experience severe food poverty compared to those from the richest households. This underscores the need for nutrition‐sensitive social protection programmes to prioritize improving access to nutritious foods among the most vulnerable populations.
- Children experiencing severe food poverty consume fewer nutrient‐rich foods and increasingly consume unhealthy, energy‐dense, nutrient‐poor foods, implying that the food system is double‐failing children's nutritional needs.

## 1 Introduction

Adequate nutrition is necessary for well‐being, normal growth, and brain development in early childhood (UNICEF 2024). The first 1000 days, from conception to the child's second birthday, represent a critical period for child growth and development. Proper nutrition during this period lays the foundation for cognitive, motor and socio‐emotional skills that shape outcomes across the life course. In contrast, nutritional deficiencies associated with monotonous diets have been linked to poor growth, which in turn is linked to impaired cognitive development, reduced productivity, and well‐being later in life (Prado and Dewey 2014).

More recently, UNICEF introduced the concept of child food poverty, which drives from dietary diversity score metrics, which are used to classify child food poverty, specifically identifies children consuming two or fewer food groups as living in severe food poverty, a threshold that carries distinct physiological and developmental implications compared to standard dietary diversity scores. Those consuming 3–4 food groups are considered to be in moderate child food poverty. Although minimum dietary diversity (MDD) answers whether a child's diet reaches a minimum acceptable level of diversity, child food poverty indicates how far below that minimum standard a child's diet falls, making it especially useful for policy, targeting, and advocacy. Globally, an estimated 440 million children under 5 years of age live in conditions of food poverty, with disproportionate burdens in low‐income countries like Ethiopia (UNICEF 2024). The national surveys from Ethiopia highlight that despite some progress, child stunting remains a serious public health concern (38%), and dietary diversity is extremely low, with only 13.8% of children (6–23 months) meeting the MDD (EDHS 2019). Furthermore, the recent Ethiopian Health and Demographic Survey illustrated that only 16 percent of children aged 6–23 months are fed with a minimum dietary (EDHS 2025). These findings underscore that inadequate diets continue to be a key driver of malnutrition and must be addressed to achieve substantial progress in its prevention.

While household food insecurity has been studied extensively, far less is known about the specific prevalence and severity of child food poverty, a measure that focuses on the quality and diversity of diets at the individual child level. This distinction is important, as children may experience food poverty even in households that are not classified as food insecure. Moreover, evidence on the magnitude, distribution, severity and factors of child food poverty in Ethiopia remains limited, particularly at the subnational level. Recent evidence also suggests that severe and moderate food poverty may have distinct consequences and potentially different factors, highlighting the importance of examining both. A clearer understanding of these factors is critical for designing effective, timely, and targeted interventions that address not only immediate nutritional needs but also underlying socio‐economic and health system drivers. To this end, this study aims to: (1) estimate the prevalence of severe and moderate child food poverty at national and regional levels; (2) identify key socio‐demographic, economic and health‐related factors; and (3) quantify the absolute number of children under five affected. The findings are intended to inform multi‐sectoral policies and programmes aimed at transforming food systems for children, improving young children's diets, and advancing Ethiopia's nutrition security agenda.

## 2 Methods

### 2.1 Study Design, Area, and Period

Data were used from the National Food and Nutrition Strategy Baseline Survey (FNSBS) for this study. The FNSBS is a population‐based cross‐sectional survey across 10 regions and two city administrations of Ethiopia. Data collection occurred in phases between July 2021 and February 2024, for details refer (Woldeyohannes et al. 2023).

### 2.2 Sampling Methods

#### 2.2.1 Sample Size Determination

The total sample size of the survey was 16,596 households, and targeted women of reproductive age, children 0–59 months, school‐aged children (6–12 years), and adolescent girls (10–19 years). The survey is nationally and regionally representative. More details about the sampling methods can be found elsewhere (Woldeyohannes et al. 2023). The present study focused on children under 0–59 months (*n* = 6187) and children under 2 years of age (*n* = 2969).

#### 2.2.2 Sampling Procedures

A two‐stage stratified cluster sampling procedure was used to select households. A total of 639 enumeration areas (EAs) (382 urban and 257 rural EAs) were selected independently in each sampling stratum with probability proportional to EA size. In each of the selected EAs, a household listing was carried out, and these lists were used as a sampling frame for the second stages of household selection. All children aged 6–59 months with in the 26 households and WRA (15–49 months) in 13 households were included in the dietary assessment. To ensure that the survey precision is comparable across regions, the sample allocation was done through a power allocation between regions (Woldeyohannes et al. 2023).

#### 2.2.3 Data Collection Tools and Procedures

The Woldeyohannes et al. (2023) employed three structured questionnaires to collect data on household characteristics, nutrition‐sensitive and specific interventions, dietary intake, and biomarkers. Tools were translated into major local languages (Amharic, Somali, Afar, and Oromiffa) and administered digitally using Open Data Kit (ODK).

### 2.3 Variable Measurement

Primary outcome variable is child food poverty. The minimum acceptable diet (MAD) is a composite indicator that combines MDD and minimum meal frequency (MMF). While this composite measure is useful for summarising overall feeding adequacy, shortfalls in child diets (child food poverty) are most often driven by poor dietary diversity rather than meal frequency. In practice, a child may be fed frequently but still consume a nutritionally inadequate, monotonous diet. For this reason, greater emphasis is placed on MDD rather than MAD, as dietary diversity is the more decisive component for identifying meaningful gaps in young children's diet.

**Factors.**

- Socio‐demographic and socio‐economic factors: Age of child, sex of child, birth order, maternal age, marital status, maternal educational level, residence and wealth index.
- Reproductive health and health service‐related factors: Number of live births, birth size, and ANC follow‐up,
- Household and community‐related factors: Family size and number of children.
- Water sanitation and hygiene: Access to pure water and sanitation.

### 2.4 Data Quality Assurance and Monitoring

Data quality procedures are detailed elsewhere (Woldeyohannes et al. 2023). Briefly, supervisors verified daily completeness and accuracy before uploading to the EPHI server. Built‐in ODK checks (range, consistency, skip patterns) minimised entry errors, and GPS tracking monitored team movements. EPHI data managers reviewed submissions and communicated corrections, while weekly calls with investigators, coordinators, and supervisors addressed field challenges and corrective actions.

### 2.5 Data Processing and Analysis

Descriptive statistics and multivariate regression analyses were computed using STATA (Version 14.0, StataCorp LLP, Texas, USA). Survey weights were applied for descriptive statistics. Multivariable logistic regression analyses were used to explore the predictors of severe child food poverty as an outcome and independent variables, including age of children, mothers' age, educational status of mothers, sex, residence, wealth quintiles, ANC visit, sanitation, access of improved and unimproved water and child's size at birth. Following multivariable logistic regressions, the residuals normal distribution was checked. To generate a valid estimation of exposure effects, only variables with *p*‐value < 0.20 in the binary logistic regression analysis were kept for multivariable logistic regression analysis. Multicollinearity was computed and the variables with variance inflation factors (VIF) < 2 were included, and a multivariable logistic regression model was applied to identify the predictor of severe child food poverty (Maldonado and Greenland 1993). The goodness of fit for multivariable logistic regression was measured with the Hosmer–Lemeshow test. All statistical analyses considered differences with *p* < 0.05 to be statistically significant.

### 2.6 Ethical Considerations

Ethics approval (Ref No. EPHI‐IRB‐317‐2020) was obtained from the institutional review board (IRB) of the Ethiopian Public Health Institute. Relevant authorities, from the federal to kebele levels, were informed, and official permission letters were obtained before the start of data collection. Written informed consent was obtained from all adult participants and from caregivers of children under five. The confidentiality and anonymity of all respondents were strictly maintained. All study procedures were conducted in accordance with the principles of the Declaration of Helsinki.

## 3 Results

### 3.1 Participant Characteristics

The analysis included 2969 children aged 6–23 months with a balanced distribution across age groups; 48% were female. Most mothers (52%) were aged 20–29 years, and 54% had no formal education. The majority of households were in the lower wealth quintiles, with 65% residing in rural areas (Table 1).

**Table 1:** Prevalence and burden of children less than two years of age living in severe and moderate child food poverty, by region, Ethiopian Food and Nutrition baseline survey.

			Sever child food poverty (0–2 food group consumed)	Moderate child food poverty (3–4 food groups consumed)	National child food poverty	Children meet minimum dietary diversity (≥ 5 food groups)
Region	Unweighted	Weighted	%	%	%	(%)
Tigray	208	220226	62	32	94	6
Afar	219	35805	50	49	99	2
Amhara	265	908867	50	44	94	6
Oromia	356	1866992	43	50	93	7
Somali	359	219706	75	23	98	2
Benishangul‐Gumuz	244	42805	37	38	75	25
SNNP	279	599032	52	40	92	8
Sidama	196	143264	48	41	89	11
Gambela	251	17570	50	45	95	5
Harari	206	12705	43	41	84	16
Addis Ababa	170	134527	19	50	69	31
Dire Dawa	216	17586	48	41	89	10
Total	2969	4,219,084	48	44	**92**	**8**

### 3.2 Prevalence and Regional Distribution of Child Food Poverty

**Figure 1:**
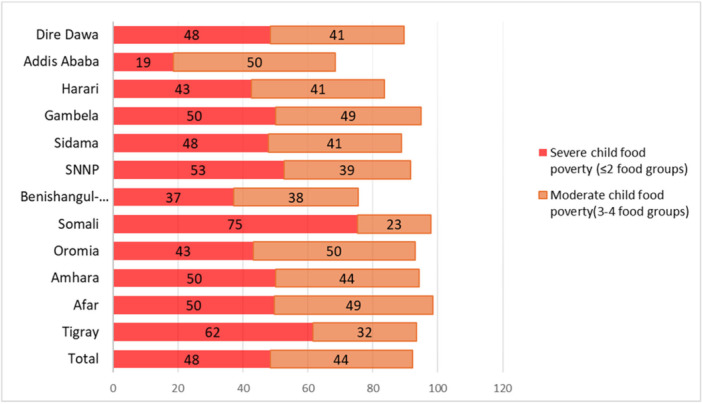
Percentage of children living in severe child food poverty and moderate child food poverty, by region.

An estimated 12 million Ethiopian children under five (92%) experience moderate or severe forms of child food poverty (Supporting Information S1: Table 1). Only 8% of children met the MDD, defined as consuming at least five of the eight recommended food groups, with higher proportions in Addis Ababa (31%) and Benishangul‐Gumuz (25%) (Table 1). Child food poverty affects all regions, but its burden varies geographically. Somali, Tigray, Afar, Amhara, Oromia, and Gambela regions show particularly high prevalence. Over 75% of children in Somali, 60% in Tigray, and 50% in Amhara live in severe child food poverty, compared to 19% in Addis Ababa (Figure 1).

Weighted estimates in under 2 years children indicate that approximately 3.61 million children consume insufficiently diverse diets, including 1.88 million in severe child food poverty, consuming diets comprising at most two food groups, and 1.73 million in moderate food poverty (Table 2).

**Table 2:** Percentage of children consuming food groups, by type and child food poverty status.

Food group	Unweighted	Weighted	Severe child food poverty (≤ 2 food groups)	Moderate child food poverty(3–4 food groups)	Child meet the minimum dietary diversity (5–8 food groups)
			Row %	Row %	Row %
Breastmilk	2366	3,267,744	42.4	49.2	8.4
Grains, roots and tubers	2105	2,978,110	32.8	57.1	10.1
Legumes, nuts and seeds	637	1,039,583	8	73.3	18.8
Dairy products (milk, infant formula, yogurt, cheese)	1138	1,421,376	27.2	57.5	15.3
Flesh foods (e.g. meat, fish, poultry, organ meats)	232	143,434	4.7	51.2	44.1
Eggs	435	480,818	9	53.2	37.9
Vitamin A‐rich fruits and vegetables	558	738,959	7.8	60.4	31.8
Other fruits and vegetables	411	432,552	6.7	48.3	44.9
Total	2969	3,930,161	48	44.2	7.7

### 3.3 Burden by Region, Residence, and Wealth

**Figure 2:**
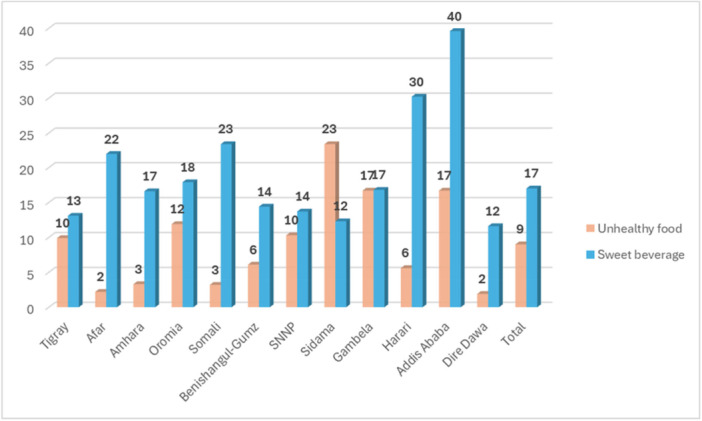
Percentage of children living in severe child food poverty who consumed an unhealthy food and sweet beverage by Region, Ethiopian Food and nutrition strategy survey.

About 75% of children living in child food poverty reside in Oromia, Amhara and Southern Nations, Nationalities, and Peoples' (SNNP) regions (Supporting Information S1: Table 1). Severe child food poverty is more prevalent in rural areas (53%) than in urban areas (36%) (Supporting Information S1: Figure 1). No significant differences were observed between boys and girls (Supporting Information S1: Figure 2).

**Figure 3:**
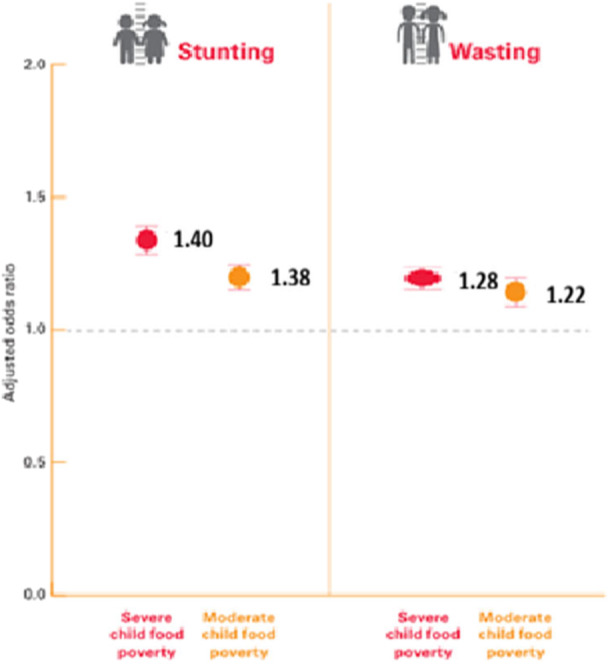
Adjusted odds ratio of child stunting and wasting by severe and moderate child food poverty in Ethiopian Children. Figure 3 shows adjusted odds ratios; the model is adjusted for child age, sex, maternal age, maternal education, household wealth quintiles, size of child during delivery, maternal access to antenatal care, residence, household sanitation and water source. The reference group is children not living in child food poverty.

Among under 2 years children the estimated 2 million children in severe food poverty, 63% belong to the poorest wealth quintiles, while only 28% come from the richest quintiles. Severe child food poverty affects 53% of children in poorer households compared to 45% in richer households (Supporting Information S1: Figure 3). The odds of living in severe food poverty are 2.6 times higher for children in the poorest households than those in the wealthiest. This wealth disparity persists across all regions except Sidama, Addis Ababa, and Benishangul‐Gumuz. This implies that severe child food poverty is experienced by children belonging to poor and non‐poor households, indicating that household income is not the only driver of child food poverty. Moderate food poverty, however, is more evenly distributed across wealth quintiles. The highest prevalence of severe food poverty among the poorest households was found in Tigray (66%), Afar (58%), and Somali (60%) regions (Supporting Information S1: Figure 4).

### 3.4 Dietary Patterns by Child Food Poverty Status

Children living in severe child food poverty consumed markedly fewer nutrient‐rich foods. Only 4% were fed flesh foods, 8% consumed vitamin A‐rich vegetables, and fewer than 10% ate legumes, eggs, or fruits (Table 2). By contrast, children meeting MDD had significantly higher consumption rates of these foods—for example, flesh foods (44%), eggs (38%), and vitamin A‐rich foods (32%) (Table 2).

Unhealthy foods (i.e., foods high in sugar, salt and/or unhealthy fats) and sweet beverages are consumed by an alarming percentage of children living in severe child food poverty. Alarmingly, 9% of children in severe food poverty consumed unhealthy foods high in salt, sugar, and oil, with the highest intake reported in Sidama (23%), Gambela (17%), and Addis Ababa (17%). Additionally, 17% consumed sugar‐sweetened beverages, notably 40% in Addis Ababa and 30% in Harari (Figure 2).

### 3.5 Factors of Severe Child Food Poverty

Multivariable logistic regression identified maternal education, antenatal care (ANC) visits, and household wealth as significant predictors of severe child food poverty (Table 3). Children of mothers with no formal education had 1.7 times higher odds (AOR 1.72; 95% CI: 1.22, 2.41) of severe food poverty compared to those with mothers having secondary or higher education. Children whose mothers had fewer than four ANC visits also had increased odds (AOR 1.70; 95% CI: 1.15,1.36) of severe food poverty. Compared to the wealthiest quintile, children from poorer and rich (but not richest) households had higher odds of severe child food poverty. No significant associations were found for child sex, rural/urban residence, access to improved water or sanitation, maternal age, or child size at birth.

**Table 3:** Multivariable logistic regression on Severe Child food poverty (< = 2 food groups) in Ethiopian Children.

Predictor variables	AOR	Std. err.	*z*	*p*‐value	[95% Conf. Interval]	
Age in months (mean)	0.97	0.01	–3.84	0.00	0.95	0.98
Sex						
Male	Ref					
Female	1.05	0.08	0.66	0.51	0.90	1.23
Age of the mother						
15–19	0.92	0.26	–0.31	0.75	0.53	1.59
20–29	0.87	0.19	–0.63	0.53	0.56	1.35
30–39	0.89	0.20	–0.53	0.60	0.57	1.38
> = 40	Ref					
Mothers' education						
No education	1.72	0.30	3.09	0.00	1.22	2.41
Primary	1.54	0.25	2.64	0.01	1.12	2.13
Secondary	1.28	0.22	1.41	0.16	0.91	1.80
More than Secondary	Ref					
Child birth size						
Large	Ref					
Average	1.00	0.12	–0.03	0.98	0.79	1.26
Small	1.03	0.15	0.22	0.83	0.77	1.38
Don't Know	1.07	0.19	0.39	0.70	0.76	1.50
ANC						
Yes	Ref					
No	1.69	0.18	5.02	0.00	1.38	2.08
Wealth quintile						
Poorest	2.94	0.70	4.57	0.00	1.85	4.68
Poorer	2.34	0.53	3.73	0.00	1.50	3.66
Middle	2.26	0.49	3.77	0.00	1.48	3.44
Richer	2.07	0.29	5.24	0.00	1.57	2.71
Richest	Ref					
Residence						
Urban	Ref					
Rural	0.86	0.15	–0.91	0.36	0.61	1.19
Drinking water						
Improved	Ref					
Unimproved	1.22	0.14	1.81	0.07	0.98	1.52
Sanitation						
Non‐open defecation	Ref					
Open defecation	1.16	0.11	1.58	0.11	0.97	1.39

Ref: means reference.

### 3.6 Child Food Poverty and Undernutrition

Severe child food poverty was associated with a higher risk of undernutrition. Children experiencing severe food poverty had 40% greater odds of stunting (AOR 1.40; 95% CI 1.12,1.49) and 28% greater odds of wasting (AOR 1.28; 95% CI 1.15,1.36) compared to children not experiencing food poverty. Moderate child food poverty also increased odds of stunting (38%) and wasting (22%) relative to no food poverty (Figure 3).

## 4 Discussion

This study offers one of the most comprehensive assessments to date of child food poverty in Ethiopia. Nearly all children under two (92%) live in moderate or severe food poverty, equating to over 12 million under‐fives, and nearly half face severe deprivation. The consequences of food poverty vary significantly by severity. Severe food poverty defined by UNICEF as children consuming two or fewer food groups is strongly associated with acute malnutrition (wasting) and immediate mortality risks. In contrast, moderate food poverty (consuming 3–4 food groups) is more closely linked to chronic malnutrition (stunting) and hidden hunger, where caloric intake may be sufficient but essential micronutrients like Vitamin A, iron, and zinc are insufficient. Research indicates that children in severe food poverty often lack animal‐source proteins and dairy, leading to irreversible deficits in cognitive development and physical growth that are more pronounced than those in moderate food poverty. Therefore, separating these categories allows for targeted public health interventions (WHO and UNICEF 2021).

The burden in Ethiopia is approximately 1.6 times higher than the Eastern and Southern Africa regional average (Eastern and Southern Africa child food poverty ~30%) (UNICEF 2024).

Child food poverty is widespread across all regions, yet the burden is uneven. Somali, Tigray, Afar, Amhara, Oromia and Gambela endure the highest levels, with three in four children in Somali living in severe food poverty. Addis Ababa, by contrast, shows the lowest prevalence, reflecting better market access and higher incomes. Indeed, the association between income, market access and diet quality has been consistently reported in earlier studies (Usman and Callo‐Concha 2021). The clustering in crisis‐affected and marginalised regions corresponds with recent nutrition and food security reports documenting repeated shocks and livelihood disruptions (WFP 2023).

Rural–urban disparities further emerge: more than half of rural children experience severe food poverty, versus just over one‐third in urban settings. This aligns with national surveys showing limited dietary diversity in rural households (EDHS 2019; EDHS 2025). Based on recent systematic reviews, the prevalence of MDD among children aged 6–23 months in Ethiopia is remarkably low, with pooled estimates often ranging between 18% and 26.7%. Only about one‐quarter of children consume the recommended minimum food groups daily, with significant disparities favouring urban over rural areas (Mulatu et al. 2024).

Subsistence farming, seasonal fluctuations, and lower market access undermine food availability in rural areas, while urban households, despite better market access, may still face affordability constraints (Sibhatu and Qaim 2018; FAO 2023; Tadesse and Shively 2013).

Socioeconomic inequalities are pronounced. Children from the poorest households are 2.6–3 times more likely to experience severe child food poverty compared to those in the wealthiest households. This mirrors global evidence that maternal education, household income and access to health and nutrition services strongly influence diet quality (Headey et al. 2018; Hirvonen et al. 2017; Tefera et al. 2021). Income inequality remains a pervasive driver, even in ostensibly food‐secure households (French et al. 2019; Rahman and Chowdhury 2007).

Dietary patterns paint a troubling picture: < 25% of children in severe food poverty consume dairy; < 20% receive breastmilk; < 10% consume legumes, eggs, or vitamin A‐rich fruits and vegetables; and < 5% consume meat. Concurrently, 10% of these children are exposed to unhealthy, ultra‐processed foods high in fat, sugar, or salt, particularly in Sidama, Gambela and Addis Ababa. This displacement of nutrient‐rich foods by energy‐dense, nutrient‐poor items is a growing concern mirrored in Nepal (Tizazu et al. 2024; Pries et al. 2019; UNICEF 2024).

Critically, children in moderate or severe food poverty face significantly higher odds of stunting (38%–40%) and wasting (22%–28%) than non‐affected children and above the national average (11%), paralleling global associations between child food poverty and undernutrition (UNICEF 2024).

Notably, no gender differences in child food poverty were observed, suggesting that feeding inequities are not primarily driven by cultural gender biases. However, structural poverty, limited assets and market disruption continue to undermine dietary access, especially in conflict‐affected regions like Tigray, Afar, and Amhara, as shown in regions like the Gaza Strip, Somalia and Ukraine, hostilities have collapsed food and health systems. In Gaza, 9 out of 10 children are surviving on two or fewer food groups per day (UNICEF 2024).

### 4.1 Strengths and Limitations

A major strength of this study is the use of nationally representative survey data, enabling robust estimates across all regions and population subgroups. The analysis applied standardised definitions of severe and moderate child food poverty, facilitating comparability with global estimates and other country contexts. Furthermore, the disaggregation by wealth quintile, place of residence, and region provides actionable insights for policy and programme design.

However, some limitations should be noted. First, the cross‐sectional nature of the data limits the ability to infer causal relationships between socio‐economic status, residence, and child food poverty. Second, dietary data were based on caregiver recall of foods consumed the previous day, which may be subject to recall and reporting bias. Third, seasonal variations in food availability and dietary patterns were not captured, which could affect prevalence estimates, particularly in agrarian regions with marked seasonal food insecurity. Finally, while the analysis identifies associations with wealth and residence, other important factors such as food environment, and market access were not examined in depth and warrant further investigation, which helps to validate and extend the findings of this analysis using longitudinal cohort studies, it is a prospective studies following children from birth through the first 24 months of life are necessary to observe how feeding patterns evolve. Such research would allow for the assessment of how changes in household food security or maternal employment status directly precede or follow shifts in dietary diversity and meal frequency.

## 5 Conclusion

This study highlights the urgent need to address severe and moderate child food poverty in Ethiopia, with a focus on reducing geographic and socio‐economic inequalities.

To reduce child food poverty, bridge equity gaps, and reach vulnerable populations, we recommend the following: an integrated, multisectoral response is urgently needed, aligned with the UNICEF First Foods Africa initiative, to reshape food systems so they support young children's nutrition:

- **Invest in local food production** by supporting the sustainable diversification of local agricultural systems, particularly focusing on animal‐source foods, fruits, and vegetables, and ensure alignment and integration with national frameworks and large‐scale programmes such as the expansion of the Seqota Declaration to effectively transform food systems for children's nutrition.
- **Boost access to nutrient‐rich foods** through local innovations, including egg powder, fish powder, and fortified multi‐ingredient flours (e.g., *Mitten*).
- **Strengthen social protection linkages to nutrition outcomes**: Expand and adapt programmes like the Productive Safety Net Programme (PSNP) to be explicitly nutrition‐sensitive, ensuring that cash or food transfers are tied to improved access to diverse, nutrient‐rich foods for households with young children.
- **Promote behaviour change,** promoting context‐specific recipes and cooking demonstrations, mass campaigns using local, seasonally available foods, and targeted counselling approaches.

By coupling targeted interventions with system‐wide reforms, Ethiopia can make meaningful progress in eliminating child food poverty, a necessary step toward breaking intergenerational cycles of malnutrition and securing a healthier, more productive future for all children.

## Author Contributions

Meron Girma, Alemnesh Petros, Meseret Woldeyohannes, Nahom Tefera, and Masresha Tessema designed and conducted the research. Bedassa Tesseam analysed the data. Adamu Bealy, Yetayesh Maru, Stanley Chitekwe, Nardos Birru, Hiwot Darsene, Kaleab Baye, and Ramadhani Noor wrote the manuscript. All authors reviewed the manuscript, made final edits, and approved the final version.

## Conflicts of Interest

The authors declare no conflicts of interest.

## Supporting information


**Supporting Figure 1:** Percentage of children living in severe child food poverty and moderate child food poverty, by Residence, Ethiopian Food and Nutrition baseline survey.

**Supporting Figure 2:** Percentage of children living in severe child food poverty and moderate child food poverty by Sex.

**Supporting Figure 3:** Percentage of children living in severe child food poverty and moderate child food poverty, by Wealth quintiles.

**Supporting Figure 4:** Percentage of children living in severe child food poverty by region, Wealth quintiles.

**Supporting Table 1:** Absolute number of under‐five children living in severe and moderate child food poverty estimated by extrapolation, by region.

## Data Availability

The data that support the findings of this study are available upon request from the corresponding author following an embargo period to allow for the conclusion of the research and publication of findings.
