# Supplementary material for: Prevalence and Drivers of Child Food Poverty in Ethiopia: Evidence From a Nationally Representative Survey
Source: Matern Child Nutr. 2026 Apr 13;22(2):e70186. doi: 10.1111/mcn.70186 (PMC13076921; doi:10.1111/mcn.70186)
Supplement: Supplementary file 3 — Supporting Figure 3: Percentage of children living in severe child food poverty and moderate child food poverty, by Wealth quintiles. [file MCN-22-e70186-s005.docx]

Supplementary Figure 3: Percentage of children living in severe child food poverty and moderate child food poverty, by Wealth quintiles,
