# Supplementary material for: Prevalence and Drivers of Child Food Poverty in Ethiopia: Evidence From a Nationally Representative Survey
Source: Matern Child Nutr. 2026 Apr 13;22(2):e70186. doi: 10.1111/mcn.70186 (PMC13076921; doi:10.1111/mcn.70186)
Supplement: Supplementary file 4 — Supporting Figure 4: Percentage of children living in severe child food poverty by region, Wealth quintiles. [file MCN-22-e70186-s004.docx]

Supplementary Figure 4: Percentage of children living in severe child food poverty by region, Wealth quintiles.

Addis Ababa

Afar

Amhara

Benishangul-Gumz

Dire Dawa

Gambela

Harari

Oromia

SNNP

Sidama

Somali

Tigray

Total

0

10

20

30

40

50

60

70

80

90

100

Percentage (%)

Wealthiest

Poorest
