# Supplementary material for: Prevalence and Drivers of Child Food Poverty in Ethiopia: Evidence From a Nationally Representative Survey
Source: Matern Child Nutr. 2026 Apr 13;22(2):e70186. doi: 10.1111/mcn.70186 (PMC13076921; doi:10.1111/mcn.70186)
Supplement: Supplementary file 5 — Supporting Table 1: Absolute number of under‐five children living in severe and moderate child food poverty estimated by extrapolation, by region. [file MCN-22-e70186-s002.docx]

Supplementary Table 1, Absolute number of under-five children living in severe and moderate child food poverty estimated by extrapolation, by region,

| **Region** | **Unweighted** | | **Weighted** | **Severe child food poverty (≤2 food groups)** | **Moderate child food poverty (3-4 food groups)** | | **National child food poverty** | | **Children meet minimum dietary diversity (> food groups)** |  |
| --- | --- | --- | --- | --- | --- | --- | --- | --- | --- | --- |
|  |  |  |  | Freq | | Freq | Freq | Freq | | |
| **Tigray** | | 701 | 737,642 | 457,338 | | 236,045 | 693,384 | | 44,259 | |
| **Afar** | | 952 | 147,252 | 91,296 | | 47,121 | 138,417 | | 8,835 | |
| **Amhara** | | 749 | 2,557,706 | 1,585,778 | | 818,466 | 2,404,244 | | 153,462 | |
| **Oromia** | | 1,096 | 5,767,961 | 3,576,136 | | 1,845,747 | 5,421,883 | | 346,078 | |
| **Somali** | | 1,274 | 782,058 | 484,876 | | 250,258 | 735,134 | | 46,923 | |
| **Benishangul-Gumuz** | | 688 | 120,987 | 75,012 | | 38,716 | 113,728 | | 7,259 | |
| **SNNP** | | 873 | 1,868,917 | 1,158,729 | | 598,053 | 1,756,782 | | 112,135 | |
| **Sidama** | | 686 | 506,680 | 314,142 | | 162,138 | 476,279 | | 30,401 | |
| **Gambela** | | 678 | 49,048 | 30,410 | | 15,695 | 46,105 | | 2,943 | |
| **Harari** | | 631 | 38,446 | 23,837 | | 12,303 | 36,139 | | 2,307 | |
| **Addis Ababa** | | 513 | 396,627 | 245,909 | | 126,921 | 372,829 | | 23,798 | |
| **Dire Dawa** | | 662 | 58,233 | 36,104 | | 18,635 | 54,739 | | 3,494 | |
| **Total** | | **9,503** | **13,031,556** | **8,079,564** | | **4,170,098** | **12,249,662** | | **781,893** | |
